# Using Telemedicine to Diagnose Surgical Site Infections in Low- and Middle-Income Countries: Systematic Review

**DOI:** 10.2196/13309

**Published:** 2019-08-19

**Authors:** Charlotte E J Sandberg, Stephen R Knight, Ahmad Uzair Qureshi, Samir Pathak

**Affiliations:** 1 Faculty of Health Sciences University of Bristol Bristol United Kingdom; 2 Centre for Informatics Usher Institute Edinburgh United Kingdom; 3 Department of Surgery Services Institute of Medical Sciences Lahore Pakistan; 4 Bristol Centre for Surgical Research Population Health Sciences Bristol Medical School Bristol United Kingdom; 5 NIHR Bristol Biomedical Research Centre Bristol United Kingdom; 6 University Hospitals Bristol NHS Foundation Trust Bristol United Kingdom

**Keywords:** surgical site infection, wound infection, developing country, low- and middle-income countries, telemedicine, postoperative, follow-up

## Abstract

**Background:**

A high burden of preventable morbidity and mortality due to surgical site infections (SSIs) occurs in low- and middle-income countries (LMICs), and most of these SSIs occur following discharge. There is a high loss to follow-up due to a wide geographical spread of patients, and cost of travel can result in delayed and missed diagnoses.

**Objective:**

This review analyzes the literature surrounding the use of telemedicine and assesses the feasibility of using mobile phone technology to both diagnose SSIs remotely in LMICs and to overcome social barriers.

**Methods:**

A literature search was performed using Medline, Embase, CINAHL, PubMed, Web of Science, the Cochrane Central Register of Controlled Trials and Google Scholar. Included were English language papers reporting the use of telemedicine for detecting SSIs in comparison to the current practice of direct clinical diagnosis. Papers were excluded if infections were not due to surgical wounds, or if SSIs were not validated with in-person diagnosis. The primary outcome of this review was to review the feasibility of telemedicine for remote SSI detection.

**Results:**

A total of 404 articles were screened and three studies were identified that reported on 2082 patients across three countries. All studies assessed the accuracy of remote diagnosis of SSIs using predetermined telephone questionnaires. In total, 44 SSIs were accurately detected using telemedicine and an additional 14 were picked up on clinical follow-up.

**Conclusions:**

The use of telemedicine has shown to be a feasible method in remote diagnosis of SSIs. Telemedicine is a useful adjunct for clinical practice in LMICs to decrease loss to postsurgical follow-up.

## Introduction

Surgical site infections (SSIs) cause preventable morbidity and mortality. The etiology of SSIs is multifactorial and is due to various perioperative factors such as: antibiotic use, cleanliness of surgical equipment and postoperative wound care [1]. The greatest burden is seen in low- and middle-income countries (LMICs) [2], and therefore investigating factors to reduce the incidence of SSIs should be a clinical priority.

Primary prevention of infection plays a significant role in decreasing morbidity and mortality; however, complete eradication of SSIs is not possible. Therefore, to minimize the morbidity of SSIs, timely management is required. This proves challenging in LMICs, as most SSIs are diagnosed after discharge [3]. The surgical population experiences a high loss to follow-up, ranging between 32-75% [3,4,5], which increases the proportion of infections that go undetected and untreated. The wide geographic spread, as a result of poor healthcare infrastructure, is a significant factor in the high loss to follow-up. Specifically, patients report long journey times, with high associated costs and loss of income as the key reasons behind nonattendance [6].

While this highlights a wider problem surrounding access to healthcare facilities in LMICs, one practical solution to improve patient follow-up after discharge is the use of telemedicine. This is possible because LMICs have seen exponential expansion of mobile phone infrastructure in recent years, allowing for greater implementation of telemedicine [7].

Previous studies reporting the use of telemedicine in LMICs have described improvement in cancer clinic attendance rates [8] and long-term surgical follow-up of cleft palate surgeries [9]. Currently, the only systematic review available that details the use of telemedicine for surgical follow-up for SSI detection is based solely within developed countries [10].

A number of studies describe the use of telemedicine in LMICs using telephone questionnaires [3-5]; however, to the authors’ knowledge, a review comprehensively summarizing the use of telemedicine within an LMIC setting has yet to be performed. Thus, the authors aimed to review the feasibility of telemedicine for diagnosis of SSIs in LMICs and the associated financial costs.

## Methods

### Overview

**Figure 1:**
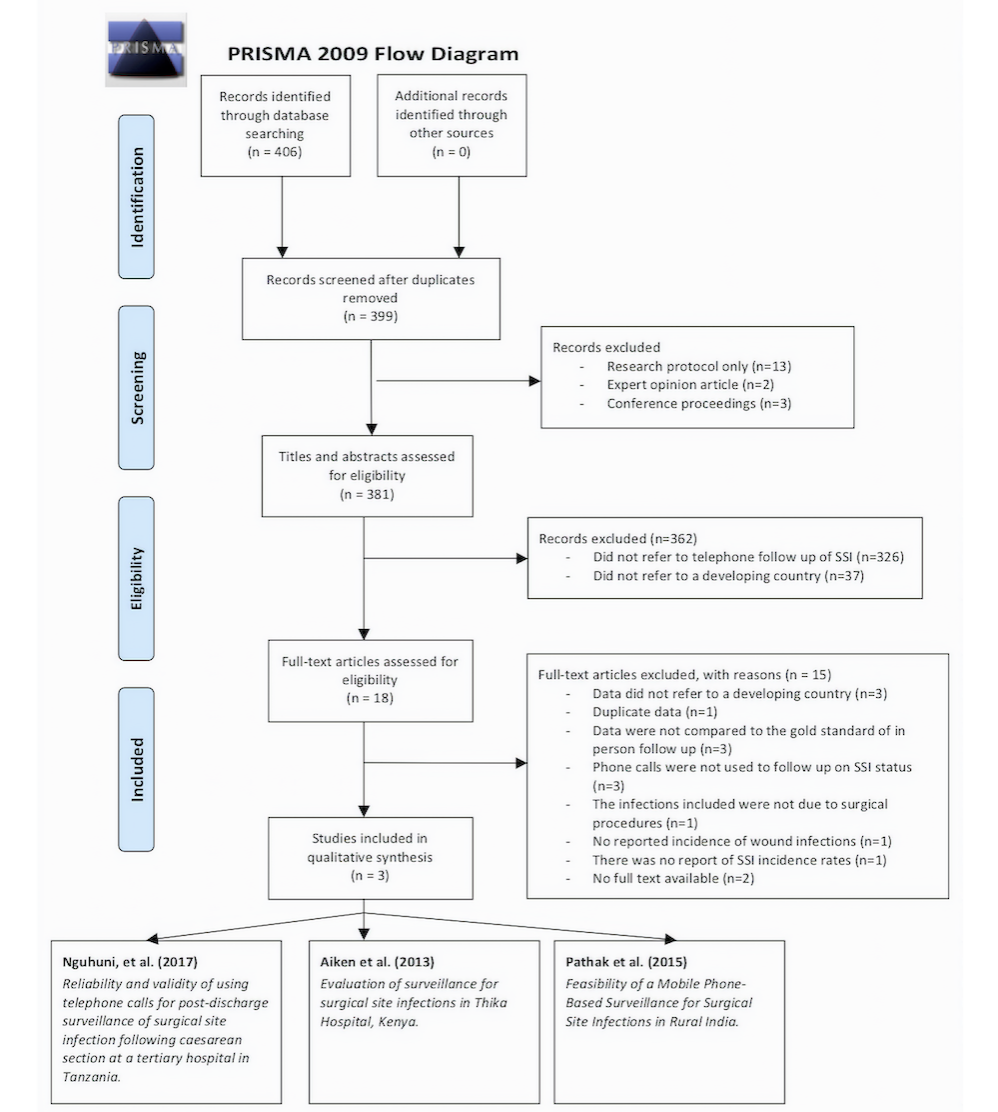
Preferred reporting items for systematic reviews and meta-analysis flow diagram.

The electronic search was conducted according to the preferred reporting items for systematic reviews and meta-analysis (PRISMA) guidelines [11] (Figure 1). The search was conducted independently by two authors (CS and SK) and included the databases Medline, Embase, CINAHL, PubMed, Web of Science, the Cochrane Central Register of Controlled Trials and Google Scholar.

MeSH search terms used were: [“developing country*” OR “LEDC” OR “resource poor setting*” OR “lower economically developed” OR “LMIC” OR “Low* and middle income” OR “low* middle income” OR “less developed” OR “global south” OR “low* income” OR “third world” OR “global health” OR “rural”] AND [“surgical site infection*” OR “surgical wound infection*” OR “SSI” OR “Surgical wound dehiscence” OR “Wound infection*” OR “Complication*” OR “Postsurgical” OR “postoperative complication*” OR “wound healing*”] AND [“phone” OR “telephone” OR “cellphone” OR “text message*” OR “phone call” OR “telephone call” OR “text” OR “telemedicine” OR “smartphone” OR “interviews as topic” OR “telephone interview*”]. Bibliographic references for each article were directly searched to identify additional studies not found in the primary electronic search. The last search date was June 2019.

### Definitions

LMICs are defined as belonging to the World Health Organization (WHO) classification of a low or low-middle income country [12] at the time of study. Low-income countries are defined as a country with a gross national income (GNI) per capita of 1025 United States Dollars (USD) or less, and low-middle income countries are defined as having a GNI per capita between 1026-4035 USD [12]. Telemedicine is defined as the use of telecommunication devices for remote delivery of medical care [13].

SSIs are defined in line with the Centers for disease control and prevention (CDC) criteria [14], stipulating infection must have occured within 30 days of primary incision and can only involve the skin and soft tissues surrounding the incision site. At least one of any of the following signs of infection must also be present: purulent discharge, positive organism cultures, heat, erythema, local edema or pain.

### Inclusion Criteria

Literature reviews and original research published in English in peer-reviewed journals were eligible for inclusion. Studies included reported the use of telephone communication for detection and diagnosis of SSIs, in comparison to the current practice of direct clinical diagnosis. All operations requiring a skin incision and overnight stay were included. All types of anesthesia were included.

### Exclusion Criteria

Animal studies, case reports, grey literature, trial protocols, editorials and expert opinion articles were excluded. Two papers were excluded due to full texts being unavailable. Conference reports and proceedings were excluded due to the high probability of incomplete data. Articles not in the English language were also excluded. Studies where telemedicine was not validated by in-person physician diagnosis were excluded. Similarly, studies that did not report SSI incidence rate or did not report on infections from surgical wounds were excluded. In the instance that multiple studies referred to the same dataset, the publication with the most complete dataset was used.

### Outcome Measures

The primary outcome measure was the number of SSIs diagnosed using telephone interviews as a proportion of the total number of SSIs diagnosed. Secondary outcome measures included determining the proportion of patients who were able to be contacted using telephone follow-up, as well as the cost of the intervention itself for the healthcare provider and the patient.

### Data Extraction

Data were extracted independently by two researchers (CS and SK) from the included studies using a standardized *pro forma*. All differences in data extraction were highlighted and discussed between the two authors. If a consensus was not reached, SP was consulted and given the deciding vote. The data were categorized as study demographics (ie, geographical location, number of included patients, follow-up time, number of follow-ups, method of telephone use), patient demographics (ie, study population, type of surgical procedures) and infection demographics (ie, SSI diagnosis criteria, SSI incidence).

Outcome data was collected in groups relating to patient characteristics (ie, number of patients with a mobile phone, number of patients participating in the telemedicine intervention, proportion of patients who were successfully followed up with using telemedicine), SSI characteristics (ie, total number of SSIs, proportion of SSIs diagnosed using telemedicine), and economic outcomes relating to the cost of telemedicine intervention.

### Quality Assessment

Objective measurement of quality and risk of bias was independently analyzed using the Methodological index for nonrandomized studies (MINORS) criteria [15] by two authors (CS and SK). In addition, the effectiveness and ability of each study to be replicated in a wider setting was assessed using the WHO mobile health evidence and reporting assessment (mERA) checklist [16].

## Results

### Study Selection

The search criteria retrieved 404 articles, of which seven were duplicates. Following screening, a total of 18 articles were identified for full text review. Three full text articles met the inclusion criteria.

Included studies were performed in Kenya [5], India [4] (low-middle income countries) and Tanzania [3] (low income country). A total of 2082 patients were included, with 172 SSIs reported overall and a mean SSI rate of 8.75%. All studies assessed the use of telephone calls to diagnose SSIs (Table 1).

**Table 1:** Included study characteristics.

Characteristics	Aiken et al	Nguhuni et al	Pathak et al
Publication year	2013	2017	2015
Geographic location	Kenya	Tanzania	India
Number of included patients	1172	374	536
Study population	All patients undergoing surgical procedure	Obstetric patients	All patients undergoing surgical procedure
Included surgical procedures	Caesarean section, laparotomy, hernia repair, orthopedic lower limb surgery, salpingectomy, cystectomy	Caesarean section only	Hernia repair, colonic and urogenital surgery
Method of telemedicine intervention	Telephone calls with predetermined questionnaire	Telephone calls with predetermined questionnaire	Telephone calls with predetermined questionnaire
Follow-up frequency	2	3	1
Follow up performed	Days 14 & 28	Days 6, 12 & 28	Day 30
Length of follow up	30 days	30 days	30 days
SSI^a^ diagnosis criteria	CDC^b^ criteria for diagnosis of SSI	CDC criteria for diagnosis of SSI	Local SSI criteria
SSI rate	7.90%	12.0%	6.34%

^a^SSI: surgical site infection

^b^CDC: centers for disease control and prevention

**Table 2:** Summary of findings.

Findings	Aiken et al	Nguhuni et al	Pathak et al
Patients providing a telephone number, %	>90	84	100
Patients with ≥1 successful telephone follow up who had provided a telephone number, %	Not stated	87	71
Patients contacted by phone and seen in clinic, %	Not stated	73	69
Patients undergoing surgical procedure	89	484	10
Patients with SSIs^a^ that participated in telephone screening, %	Not stated	72	Not stated
SSIs detected over phone or clinic review at day 7	Not stated	11/14	Not stated
SSIs detected over phone or by clinical review at day 14	Not stated	7/11	Not stated
SSIs detected over phone or by clinical review at day 30	Not stated	0/0	Not stated
Total SSIs detected over phone or by clinical review	16 / 23	18 / 25	10 / 10

^a^SSI: surgical site infection

### Accuracy of Telephone Follow-Up Diagnosis

Of all the cases, 44 SSIs were diagnosed over the phone [3,4,5], and all were confirmed as SSIs on clinical follow-up. However, 14 additional SSIs were not detected over the phone and only diagnosed on clinical follow-up (Table 2).

### Number of Patients Able to be Contacted by Phone

A total of 172 SSIs were recorded across the three studies [3,4,5]; however, only 58 of these SSIs occurred in populations who took part in telephone follow-up. The remaining 114 SSIs were diagnosed in follow-up clinics and involved patients not contacted via telemedicine.

Within the three studies, between 84-100% of patients provided a telephone number [3,4,5]; however, 13-29% of these patients were unable to be contacted. Loss to follow-up occurred due to patients not returning to clinic after telemedicine communication. This resulted in an inability to confirm SSI diagnosis.

### Cost Analysis

Only one study [3] commented on the cost of intervention. Phone calls were recorded as lasting between 3-5 minutes, and it was estimated that each phone call cost approximately 0.50 USD. As each patient was followed up with on days 7, 14 and 28, this would be equivalent to 1.50 USD per patient (0.23% of Tanzanian gross domestic product [GDP] per capita at the time of calculation). In a reflection of the cost for in-person outpatient appointments, 2008 data supplied by the WHO documents one hospital outpatient appointment in Tanzania as costing a minimum of 1.27 USD [17], which is 2.54 times more expensive than if telephone follow up were to be used.

### Quality Assessment of Studies

Each of the studies was assessed using the MINORS criteria [15] to determine their quality. As they were all comparative prospective studies, the maximum score was 24. The individual study scores were between 14-19 (Table 3).

The MINORS criteria assess whether the paper has a clearly stated aim, if it includes all eligible patients, if it collected data according to a preestablished protocol, if there was an appropriate endpoint to the aim of the study, if there was an unbiased assessment of the study endpoint, if it had an appropriate follow-up period, whether it had a loss to follow up less than 5%, if it prospectively calculated the study size, whether or not it utilized an adequate control group, if the study group was managed during the same time period as the control, if there was a baseline equivalence of the two groups and if there is adequate statistical analysis.

When each text was compared against the WHO mERA checklist (Table 4), they scored between eight to nine points out of a possible 16. The mERA checklist requires authors to present the availability of infrastructure to support the intervention and justify its use; to describe how the intervention can integrate into existing health information systems and how this will be delivered; to describe formative research on the intervention; to provide user feedback; to provide a description of how people are informed of the program; to mention barriers to access and costs assessment; and, finally, they must evaluate the limitations of delivery at scale and of adapting the intervention to other populations.

**Table 3:** Methodological index for nonrandomized studies criteria for each paper.

Criteria	Studies					
	Aiken et al [5]		Nguhuni et al [3]		Pathak et al [4]	
	Score	Description	Score	Description	Score	Description
1^a^	2	—^b^	2	—	2	—
2^c^	2	—	1	Did not state reason some patients were excluded	1	No inclusion criteria stated
3^d^	1	References a pilot study	0	Doesn't report a pilot or protocol	0	No pilot or protocol mentioned
4^e^	2	SSIs^f^ diagnosed in reference to the standardized CDC-NHNS^g^ risk criteria	2	SSIs diagnosed in reference to the standardized CDC-NHNS risk criteria	0	No criteria for SSI diagnosis explained
5^h^	1	Not stated whether those in clinic were blinded to the telephone outcomes	1	Not stated whether those in clinic were blinded to the telephone outcomes	1	Not stated whether those in clinic were blinded to the telephone outcomes
6^i^	2	30 days	2	30 days	2	30 days
7^j^	0	The loss to follow up was not commented on	1	Loss to follow up was 13%, but 26% of those who had been contacted by phone did not return to clinic	2	No loss to follow up
8^k^	2	Specificity, sensitivity	2	Specificity, sensitivity, positive predictive values, negative predictive values	1	Not recorded
9^l^	2	Compared against clinical diagnosis	2	Compared against clinical diagnosis	2	Compared against clinical diagnosis
10^m^	2	Patients were their own control	2	Patients were their own control	2	Patients were their own control
11^n^	0	No Table 1 referenced	2	—	0	No data on group equivalence mentioned
12^o^	2	—	2	—	1	No statistics mentioned for SSI detection via mobile phone
Total score^p^	18	—	19	—	14	—

^a^Has a clearly stated aim.

^b^Not applicable/no description given.

^c^Includes all eligible patients.

^d^Collected data according to a preestablished protocol.

^e^There was an appropriate endpoint to the aim of the study.

^f^SSI: surgical site infection.

^g^CDC-NHNS: Centers for Disease Control and Prevention-National Healthcare Safety Network.

^h^There was an unbiased assessment of the study endpoint.

^i^Had an appropriate follow-up period.

^j^Had a loss to follow up less than 5%.

^k^Prospectively calculated the study size.

^l^Utilized an adequate control group.

^m^Study group was managed during the same time period as the control.

^n^Baseline equivalence of the study group and control group.

^o^Adequate statistical analysis.

^p^Out of 24.

**Table 4:** Mobile health evidence and reporting assessment checklist.

Criteria	Aiken et al [5]	Nguhuni et al [3]	Pathak et al [4]
Infrastructure (population level)	No	No	No
Technology platform	Yes	Yes	Yes
Interoperability or HIS^a^ context	Yes	Yes	Yes
Intervention delivery	Yes	Yes	Yes
Intervention content	Yes	Yes	Yes
Usability/content testing	No	No	No
User feedback	No	No	No
Access of individual participants	No	No	Yes
Cost assessment	No	Yes	No
Adoption inputs/ program entry	Yes	Yes	Yes
Limitations for delivery at scale	No	No	Yes
Contextual adaptability	No	No	No
Replicability	Yes	Yes	Yes
Data security	No	No	No
Compliance with national guidelines or regulatory statutes	No	No	No
Fidelity of the intervention	Yes	Yes	Yes
Total (out of 16)	7	8	9

^a^HIS: health information systems.

## Discussion

### Primary Findings

More operative interventions are being undertaken in LMICs, however, higher rates of postoperative complications in LMICs are seen when compared with high income countries. Among these complications, SSIs have been found to be the most common [2]. The aim of this review was to establish the feasibility of using telemedicine to increase SSI detection and reduce the associated complications.

This review demonstrates that using telemedicine is a feasible intervention. All studies reported high telemedicine access and all SSIs diagnosed using telemedicine were found to be accurate. However, telemedicine alone underdiagnosed SSIs, as additional superficial infections were picked up on during in-person clinical review.

Additionally, in terms of cost effectiveness, telemedicine intervention was found to be 2.54 times less expensive than hospital outpatient appointments, using 2008 data supplied by the WHO recording one hospital outpatient appointment in Tanzania as costing a minimum of $1.27 [17].

Furthermore, based on an extrapolation of similar study data that showed a follow-up rate of 54% [18], the use of telemedicine could have detected an additional 8.28 SSIs otherwise lost to follow-up. This amounts to 36.59 USD per disability adjusted life years (DALY), where the DALY is an undiagnosed infection. This is considered a highly cost-effective intervention as per the WHO Choosing interventions that are cost-effective (CHOICE) guidelines [19], since the intervention is less than one third of Tanzania’s 2015 GDP per capita (290.66 USD) [20]. However, as only one paper discussed cost-effectiveness, firm conclusions on the cost of telemedicine intervention as a whole cannot be extrapolated from these data alone.

The included studies were able to reach up to 87% of patients for follow-up using telemedicine. This is much higher than the reported follow-up in a comparable Tanzanian study that did not use telemedicine, where only 46% of patients returned in person to clinics [18].

These results also reflect the success of LMIC telemedicine interventions in other medical fields. One study on follow-up of cancer patients in Nigeria found an additional 78.4% of patients completed all follow-up appointments if they were contacted by phone instead of being required to return to the hospital [8]. Telemedicine has also shown good efficacy in increasing patient compliance with returning for hospital follow-up, due to increased interaction with health care providers through text and call reminders [9,21].

This study strengthens the existing evidence for the incorporation of more telemedicine services in LMICs, as a practical way of delivering healthcare in remote settings. In Tanzania, two phone calls at day 7 and day 14 would have picked up over 90% of SSIs [3], evidence that this intervention can be a useful addition to clinicians’ practices. However, across the three studies, 25% of SSIs were missed by telemedicine alone. This shows that, currently, the intervention may have a low sensitivity that can limit its application.

### Limitations

The most notable limitation of this review was that only three studies were eligible for inclusion in the final analysis. This may have been a result of only considering articles in the English language; however, this is not thought to bias overall results [22,23]. Furthermore, there was high heterogeneity between studies, due to studies being undertaken in different geographical areas, involving different surgical procedures, and in populations without standardized SSI diagnosis criteria between the three studies. As a result, meta-analysis was not considered applicable.

Additionally, within all three papers there is potential reporting bias due to a lag period of 2-7 days between phone diagnoses of SSI and clinical follow up. As SSI status is able to change within this window, this may have affected results. Furthermore, none of the studies confirmed whether diagnosing clinicians were blinded to the telephone results. There is further potential bias with regards to the subjectivity of CDC diagnosis criteria, such as whether a wound is considered erythematous or hot. Although this is explored by Nguhuni et al [3] and Aiken et al [5], Pathak et al [4] do not comment upon this.

A potential consequence of these biases is the low reported incidence of SSIs across the three studies (6.3% [4], 7.9% [5] and 12% [3]), of which 93% were reported as superficial. This is markedly lower than the SSI incidence reported in the GlobalSurg 2 study [24], which found LMICs to report an average SSI rate of 14-23.2%, with 81.1% being superficial. This discrepancy may be due to caesarean sections being the most common procedure represented within the study data, as these tend to be elective operations, with clean wounds and minimal contamination. Furthermore, patients undergoing caesarean section are not representative of the wider surgical population, as they are usually otherwise healthy young women. This limits the generalizability of the data analyzed.

Regarding the included data, while a total of 2719 patients were eligible for inclusion and up to 90% of patients provided telephone numbers [3] across the studies, only 573 patients were recorded as having successful telephone follow-ups with clinical confirmation and were therefore able to be included in the final analysis. Consequently, this may also limit the results. Moreover, telemedicine access is likely to be correlated to socioeconomic status and literacy, which was not commented upon in any of the included studies. It could therefore be argued that while telemedicine addresses the need for access to healthcare, it does so unequally, increasing social inequality.

**Figure 2:**
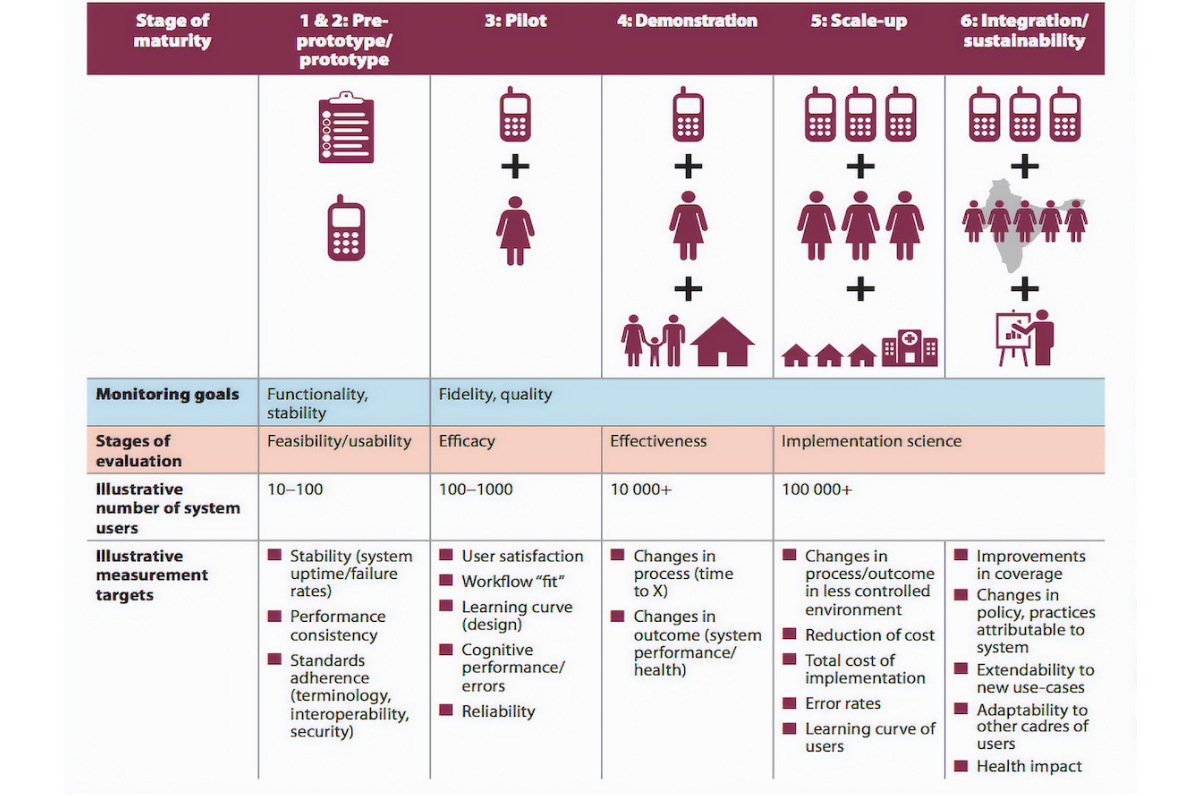
World Health Organization Mobile health evidence and reporting assessment schematic depiction of the six stages of the intervention maturity life-cycle, from preprototype to national level deployment.

None of the studies included in this review commented upon subsequent interventions or outcomes due to increased SSI detection, therefore the impact of the intervention is currently unclear. Future work should incorporate this and be undertaken using the WHO guidelines on monitoring and evaluation of digital health interventions [16] (Figure 2). Initially, pilot studies should be used to better measure the efficacy of telemedicine use, then they should be followed up by scaling-up of the intervention to measure the effectiveness of the intervention itself as well as changes in patient outcomes in both trial and less controlled environments.

Furthermore, the studies analyzed in this review only considered the use of telephone consultation; however, as mobile data access increases, ease of access to modalities such as image capture and live video streaming will also increase. This may help increase accuracy of diagnosis, as clinicians are able to simultaneously view the surgical site and question the patient. Therefore, ongoing study should not be limited to previously used telemedicine technology but instead focus on the most appropriate in terms of ease of use, accuracy of diagnosis, time and cost.

### Conclusion

The use of telephone surveillance has been shown to be a feasible option for follow-up in comparison to in-person diagnosis, with evidence that it is also cost effective. The main advantage of its use is that it can help identify SSIs that would otherwise be unreported, thus increasing the potential prompt management of time sensitive infections. However, currently, the quantitative impact of this intervention has yet to be calculated, so more research is needed to address this. Future studies should also aim to improve the accuracy of diagnosis via telemedicine through strengthening questioning methods and exploring other modalities such as live video streaming. With application of robust telephone screening programs, there is evidence that this has the potential to be a great addition to the tools used to improve the high SSI morbidity and mortality seen in LMICs.

## Abbreviations

CDC: centers for disease control and prevention
CHOICE: Choosing interventions that are cost-effective
DALY: Disability adjusted life years
GDP: gross domestic product
GNI: Gross national income
LMIC: Low-middle income country
mERA: Mobile health evidence and reporting assessment
NHS: National Health Service
NIHR: National Institute for Health Research
SSI: surgical site infection
USD: United States dollar
WHO: World Health Organization
